# CD9 Negatively Regulates CD26 Expression and Inhibits CD26-Mediated Enhancement of Invasive Potential of Malignant Mesothelioma Cells

**DOI:** 10.1371/journal.pone.0086671

**Published:** 2014-01-23

**Authors:** Toshihiro Okamoto, Satoshi Iwata, Hiroto Yamazaki, Ryo Hatano, Eriko Komiya, Nam H. Dang, Kei Ohnuma, Chikao Morimoto

**Affiliations:** 1 Department of Therapy Development and Innovation for Immune disorders and Cancers, Graduate School of Medicine, Juntendo University, Tokyo, Japan; 2 Division of Clinical Immunology, Institute of Medical Science, University of Tokyo, Tokyo, Japan; 3 Division of Hematology and Oncology, University of Florida Shands Cancer Center, Gainesville, Florida, United States of America; Wayne State University, United States of America

## Abstract

CD26/dipeptidyl peptidase IV is a cell surface glycoprotein which consists of multiple functional domains beside its ectopeptidase site. A growing body of evidence indicates that elevated expression of CD26 correlates with disease aggressiveness and invasive potential of selected malignancies. To further explore the molecular mechanisms involved in this clinical behavior, our current work focused on the interaction between CD26 and CD9, which were recently identified as novel markers for cancer stem cells in malignant mesothelioma. We found that CD26 and CD9 co-modulated and co-precipitated with each other in the malignant mesothelioma cell lines ACC-MESO1 and MSTO-211H. SiRNA study revealed that depletion of CD26 led to increased CD9 expression, while depletion of CD9 resulted in increased CD26 expression. Consistent with these findings was the fact that gene transfer of CD26 into CD26-negative MSTO-211H cells reduced CD9 expression. Cell invasion assay showed that overexpression of CD26 or gene depletion of CD9 led to enhanced invasiveness, while CD26 gene depletion resulted in reduced invasive potential. Furthermore, our work suggested that this enhanced invasiveness may be partly mediated by α5β1 integrin, since co-precipitation studies demonstrated an association between CD26 and α5β1 integrin. Finally, gene depletion of CD9 resulted in elevated protein levels and tyrosine phosphorylation of FAK and Cas-L, which are downstream of β1 integrin, while depletion of CD26 led to a reduction in the levels of these molecules. Collectively, our findings suggest that CD26 potentiates tumor cell invasion through its interaction with α5β1 integrin, and CD9 negatively regulates tumor cell invasion by reducing the level of CD26-α5β1 integrin complex through an inverse correlation between CD9 and CD26 expression. Our results also suggest that CD26 and CD9 serve as potential biomarkers as well as promising molecular targets for novel therapeutic approaches in malignant mesothelioma and other malignancies.

## Introduction

Malignant pleural mesothelioma is an aggressive malignancy arising from the mesothelial cells lining the pleura [1]. It is generally associated with a history of asbestos exposure and has a very poor prognosis [1]. In fact, the median survival is less than 12 months, with most patients dying within 10 to 17 months of their first symptoms. Moreover, the incident of malignant mesothelioma has increased in industrialized nations as a result of past widespread exposure to asbestos [2].

CD26 is a 110-kDa cell surface glycoprotein with known dipeptidyl peptidase IV (DPPIV; EC 3.4.14.5) activity in its extracellular domain [3] and is capable of cleaving N-terminal dipeptides with either L-proline or L-alanine at the penultimate position [3]. CD26 activity is dependent on cell type and the microenvironment factors that can influence its multiple biological roles [3]–[6]. Association with various proteins, including fibroblast-activation protein-α, plasminogen, adenosine deaminase, CD45 and collagen, influences its activity [3]. As a result of its various interactions, CD26 has an important, but complex, function in cellular behavior, with its biologic effect dependent on the cell type and the microenvironment.

Likely, as a result of this multifunctional characteristic, CD26 is associated with a high level of clinical aggressiveness in some tumors but a lower level in others [7], [8]. For example, it is a marker of aggressive disease for certain subsets of T-cell non-Hodgkin lymphomas/leukemias, with expression of CD26 on T-lymphoblastic lymphomas/acute lymphoblastic leukemia cells being associated with a worse outcome compared with CD26-negative tumors [9]. CD26 is also expressed at high levels on renal carcinoma cells [10]. In an immunohistochemical analysis of 152 patients with gastrointestinal stromal tumors (GIST), CD26 was found to be associated with a poorer overall survival [11]. In addition, CD26 can serve as a prognostic marker in B-cell chronic lymphocytic leukemia [12]. Furthermore, CD26 itself may be a novel therapeutic target. Anti-CD26 monoclonal antibody (mAb) treatment resulted in both *in vitro and in vivo* antitumor activity against several tumor types, including lymphoma and renal cell carcinoma [13], [14]. Our recent work showed that CD26 is preferentially expressed on malignant mesothelioma cells but not on normal mesothelial cells, and suggested that membranous expression of CD26 is of potential importance in the treatment of mesothelioma patients [15]. Importantly, humanized anti-CD26 antibody inhibited growth of malignant mesothelioma cells and induced long-term survival of tumor-transplanted immunodeficient mice [16].

CD9, a member of the tetraspanin superfamily, has been implicated in the regulation of various physiological processes, including cell motility, adhesion and fusion through an association with integrin family proteins [17]. CD9 was identified as a molecule that suppresses cellular motility and metastatic potential of a human lung adenocarcinoma cell line [18]. Clinicopathologic findings indicated that CD9 may be a predictor for better prognosis in lung adenocarcinoma [19].

While an inverse correlation between CD9 expression and metastatic development was observed in melanoma, cervical carcinoma and multiple myeloma, up-regulation of CD9 was demonstrated in aggressive gastric carcinoma [20] and high-grade astrocytic tumors [21]. The regulatory role of CD9 in cell motility therefore appears to be complex and may vary depending on the presence of an endothelial barrier and other factors.

More recently, we have identified CD9, CD24 and CD26 as cancer stem cell markers of malignant mesothelioma cells that correlated with primary stem cell signatures [22], [23]. In addition, CD26 and CD9 have been reported to be associated with several cell surface molecules such as CD45, CXCR4, M6P/IGFIIR [3], and integrin family molecules [24], respectively.

Our present study focused on the molecular association between CD26 and CD9 and found that the interaction between these two molecules plays a significant role in invasiveness, motility, and proliferation of malignant mesothelioma cells.

## Materials and Methods

### Ethics Statement

All experiments using mice were approved by and carried out following the guidelines of the Institute Animal Care and Use Committee of the University of Tokyo (Tokyo, Japan). Details of approval and animal welfare considerations are described in Checklist S1.

### Cell lines

Mesothelioma cell line ACC-MESO1 (MESO1) was obtained from RIKEN BioResource Center (Tsukuba, Japan). MSTO-211H (MSTO), NCI-H2452, and NCI-H226 were obtained from ATCC (Manassas, VA, USA). These cell lines were maintained in RPMI 1640 supplemented with 10% heat-inactivated fetal calf serum (FCS), penicillin (100 U/ml) and streptomycin (100 µg/ml) at 37°C and 5% CO_2_.

### Antibodies

Humanized anti-CD26 mAb and anti-CD9 mAb were developed in our laboratory [16], [25]. Antibodies against integrin α1(rabbit polyclonal), α2(rabbit monoclonal EPR5788) and α3(rabbit polyclonal) were purchased from Abcam (Cambridge, UK), and mouse monoclonal antibodies against integrin α4 (3G6), α5 (2H6), α6 (2C3A), and β1 (4B4) were developed in our laboratory [26]–[28]. Mouse monoclonal antibody against FAK (10G2)and rabbit polyclonal antibody against Cas-L (TA248) were developed in our laboratory. Mouse monoclonal antibody against Cas-L was obtained from ImmuQuest (Seamer, UK). Anti-phosphotyrosine (pTyr) mouse monoclonal antibody (4G10) was produced from hybridoma obtained from ATCC. Anti-CD26 goat polyclonal antibody was purchased from R&D Systems (Minneapolis, MN, USA). For flow cytometric analysis, anti-CD26-FITC, anti-CD9-FITC, anti-CD9-PE were obtained from BD Biosciences (San Jose, CA, USA), and anti-CD26-FITC (5K78)(IgM) and FITC-conjugated antibody to mouse IgM were purchased from Santa Cruz Biotechnology, Inc. (Santa Cruz, CA, USA). The secondary antibodies against goat IgG (H+L), rabbit IgG (H+L), and mouse IgG (L) conjugated with HRP were obtained from Jackson ImmunoResearch Laboratories, Inc. (West Grove, PA, USA). Human IgG andanti-actin rabbit polyclonal antibody were purchased from Sigma-Aldrich Co. LLC (St. Louis, MO, USA) and Abcam, respectively.

### Transfection of CD26 cDNA

MSTO-Wild cells were transfected with full-length cDNA of CD26 subcloned in retroviral plasmid pLNCX2 vector (Clontech, Mountain View, CA, USA) using the Lipofectamine reagent (Invitrogen, Carlsbad, CA, USA). Fresh RPMI 1640 with 10% FCS was replenished 19 h after transfection and cells were harvested after 48 h. As a control, cells were transfected with the pLNCX2 vector. Expression of the transfected cDNA was confirmed by immunoblotting and flow cytometry.

### Small interfering RNAs and short hairpin RNAs for CD26 and CD9

To deplete endogenous CD26 mRNA, three small interfering (si) RNAs and two short hairpins (sh) RNAs were obtained from Qiagen (Hilden, Germany) or Sigma-Aldrich (reference sequence: NM_001935). The sequences are as follows.

CD26 siRNA-1: 5′-ACACTCTAACTGATTACTAA-3′,

CD26 siRNA-2: 5′-CAGTAAAGAGGCGAAGTATTA-3′

CD26 siRNA-3: 5′-ATCGGGAAGTGGCGTGTTCAA-3′

CD26 shRNA-1: 5′-CCGGGACTGAAGTTATACTCCTTAACTCGAGTTAAGGAGTATAACTTCAGTCTTTTTG-3′

CD26 shRNA-2: 5′-CCGGCCAATGCAACTTCCATACAAACTCGAGTTTGTATGGAAGTTGCATTGGTTTTTG-3′

To deplete endogenous CD9, two siRNAs and two shRNAs were used (reference sequence: NM_001769). The sequences are as follows.

CD9 siRNA-1: 5′-CGTGGAACAGTTTATCTCAT-3′

CD9 siRNA-2: 5′-AATTGCCGTGGTCATGATATT-3′

CD9 shRNA-1: 5′-CCGGGCTGTTCGGATTTAACTTCATCTCGAGATGAAGTTAAATCCGAACAGCTTTTTG-3′

CD9 shRNA-2: 5′-CCGGCACAAGGATGAGGTGATTAAGCTCGAGCTTAATCACCTCATCCTTGTGTTTTTG-3′

For controls, negative control siRNA (Qiagen) or non-target shRNA control (Sigma-Aldrich) was used.

### Transfection of siRNAs and shRNAs

For assays involving siRNA, cells (3×10^4^) were cultured for 24 h on 24-well plates and transfected with 22 nmol/L siRNA cocktail against CD26, CD9 or negative control siRNA using TransIT-TKO transfection reagents (Mirus Bio LLC, Madison, WI, USA). For shRNAs, lentiviral plasmid containing each shRNA above (MISSION shRNA; Sigma-Aldrich), or non-targeting control plasmid was co-transfected with ViraPower Lentiviral packaging mix to 293FT cells using Lipofectamine 2000 (Invitrogen). The mesothelioma cell lines were infected with the shRNA-expressing lentivirus, and stable cell lines were generated by selection with puromycin.

### Flow cytometry and Fluorescence-activated cell sorting (FACS) analysis

Cells were stained with the monoclonal antibodies described above for 30 min on ice. For controls, cells were incubated with isotype-matched IgG. Cells were analyzed by BD FACSCalibur or sorted by BD FACSAria. Data were analyzed by FlowJo software (Tree Star, Inc., Ashland, OR, USA).

### Microarray analysis

MESO1 cells transfected with control siRNA or CD26 siRNA were used to examine the effect of CD26 depletion. MSTO-Wild and MSTO-CD26 (+) cells were used to study the effect of CD26 overexpression. Total RNA was isolated using TRIzol (Invitrogen) and subjected to DNA microarray analysis with DNA Chip 3D Gene (TORAY, Yokohama, Japan). A heat map of tetraspanin genes differently expressed between CD26-depleted and CD26-overexpressed mesothelioma cells was constructed by hierarchical cluster analysis using cluster 3.0 software and the results were displayed with the TreeView program. The data discussed in this publication have been deposited in NCBI's Gene Expression Omnibus and are accessible through GEO Series accession number GSE52216.

### Immunoblotting

Cells were collected and suspended in lysis buffer (50 mM HEPES, pH7.4, 150 mM NaCl, 1.0% Triton X-100, 30 mM sodium pyrophosphate, 50 mM NaF, 1 mM Na_3_VO_4_). The cell lysates were centrifuged at 15,000 rpm for 15 min at 4°C and the supernatants were stored at −80 °C or used for experiments directly. Ten micrograms of proteins were electrophoresed on SDS-PAGE and transferred to a polyvinylidene fluoride (PVDF) microporous membrane Immobilon-P (Millipore, Billerica, MA, USA). The blots were probed with the indicated antibodies, and incubated with horseradish peroxidase-conjugated antibody. Detection of proteins was carried out using ECL advance system (GEHealthcare, Wauwatosa, WI, USA).

### Immunoprecipitation

Cells (2×10^6^) were lysed in 1 ml of lysis buffer and incubated on ice for 30 min. The total cell lysates were centrifuged at 15,000 rpm for 15 min, at 4 °C and the supernatants were incubated overnight with the first antibody at 4 °C. The complexes were precipitated by adding 30 µl of protein G-agarose beads (GE Healthcare) to the lysate and incubated for 60 min at 4 °C. The beads were centrifuged at 5,000 rpm for 30 s, at 4 °C, and washed five times with ice-cold lysis buffer. The samples were suspended and denatured in SDS sample buffer (50 mM Tris pH 6.8, 2% SDS, 100 mM dithiothreitol, 10% glycerol, 0.01% bromophenol blue).

### Reverse transcription-PCR

Total RNA was isolated using TRIzol (Invitrogen). Reverse transcription-PCR (RT-PCR) was performed with total RNA which was reverse transcribed with a cDNA synthesis kit (Invitrogen) with random hexamers. PCR was performed with Takara Taq polymerase (Takara BIO). PCR primer sets were as follows:

CD26 (5′-CGGTCCTGGTCTGCCCCTCTA-3′ and 5′-CGCCACGGCTATTCCACACTT-3′)

CD9 (5′-CCGGTCAAAGGAGGCACCAAG-3′ and 5′-GATAAACTGTTCCACGCCCCC-3′)

GAPDH (5′-ACCACAGTCCATGCCATCAC-3′ and 5′-TCCACCACCCTGTTGCTGTA-3′)

### Boyden chamber-based cell invasion and migration assay

For Boyden chamber-based cell invasion assay, tumor cells (0.5×10^4^) suspended in 0.5 ml of RPMI-1640 containing 0.1% FCS were plated on Matrigel-coated 8-µm pore diameter polypropylene filter inserts in the Boyden chamber (BD Biosciences). Cells were allowed to migrate for 24 h toward 1 ml of RPMI-1640 containing 10% FCS as chemoattractant. Cells remaining in the insert were removed with a cotton swab, and the cells which attached to the bottom of the filter were stained with Diff-Quick-Staining kit (Sysmex, Kobe, Japan) and counted under optical microscope. At least five fields were counted in each experiment. For assays in the presence of antibodies, cells were pretreated with antibody at the indicated concentration for 15 min at room temperature. For Boyden chamber-based cell motility assay, uncoated inserts (8-µm pore diameter) was used.

### MTT proliferation assay

An MTT proliferation assay was performed using TetraColorONE system (SeikagakuBioscience, Tokyo, Japan). Cells (2.2×10^3^) were plated on a 96 well culture plate in RPMI 1640 containing 10% FCS. Cells were cultured and MTT proliferation assay was performed at indicated times.

### In vivo xenograft study

All experiments using mice were approved by and carried out following the guidelines of the Institute Animal Care and Use Committee of the University of Tokyo (Tokyo, Japan). Female SCID mice (5–6 weeks age) were purchased from Charles River (Yokohama, Japan). Mice were anesthetized with ether and subjected to direct s.c. inoculation of mesothelioma cells (5×10^5^ per mouse) into the dorsal region. The mice were sacrificed at day 14 after tumor cell implantation and tumors were sampled. Details of animal studies are presented in Checklist S1.

### Statistical analysis

The data are shown as the mean ± SE. The statistical significance of differences was evaluated by two-tailed *t*-test, and *P* values<0.05 was considered significant.

## Results

### Association of CD26 and CD9 in malignant mesothelioma cell lines

**Figure 1.**
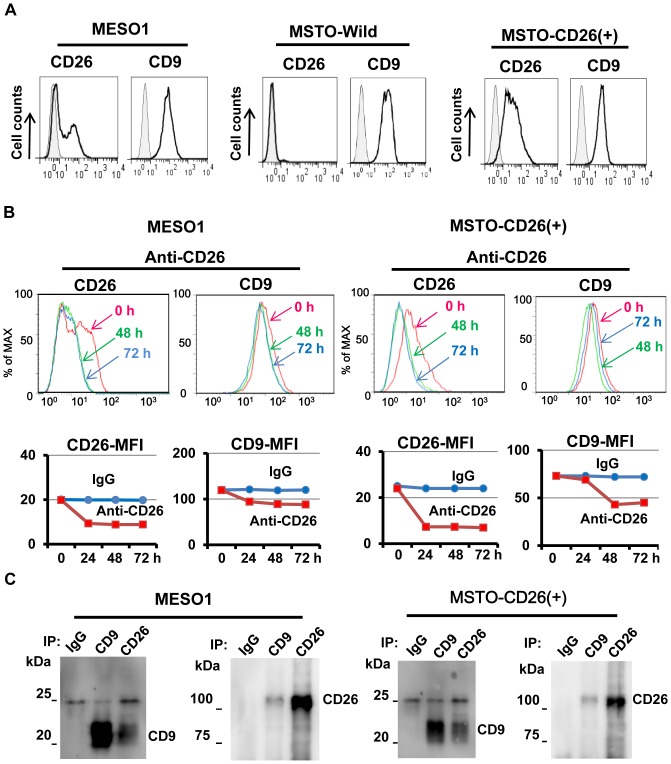
CD26 associates with CD9. (A). Flow cytometric analysis of CD26 and CD9 expression on MESO1, MSTO-Wild or MSTO-CD26 (+) cells. (B). MESO1 or MSTO-CD26 (+) cells were incubated up to 72 h at 37 °C with either control IgG (10 µg/ml) or humanized anti-CD26 mAb (10 µg/ml). These cells were stained with anti-CD26-FITC (5K76) or with anti-CD9-FITC, and subjected to flow cytometry. Intensity of modulation was indicated by mean fluorescence intensity (MFI). (C). MESO1 or MSTO-CD26 (+) cells were subjected to immunoprecipitation with control IgG, humanized anti-CD26 mAb, and anti-CD9 mAb (5H9). Immunoblot was conducted with anti-CD26 polyclonal antibody, and anti-CD9 mAb (5H9). These results were also confirmed by 5 separate experiments.

We recently reported that CD26 and CD9 are cancer stem cell markers of malignant mesothelioma [22], [23]. Since CD26 and CD9 have been reported to be associated with specific molecules [4], [24], we attempted to determine the relationship between CD26 and CD9 in malignant mesothelioma cell lines. We used the mesothelioma cell line ACC-MESO1 (MESO1) cells which naturally express CD26, MSTO-211H cells which do not express CD26 (MSTO-Wild), and CD26 transfectant of MSTO-211H cells (designated as MSTO-CD26 (+) cells) (Figure 1A).

We first analyzed antigenic modulation induced by the addition of anti-CD26 mAbin these mesothelioma cells as described previously [29]. As shown in Figure 1B, treatment with anti-CD26 mAb caused modulation of CD26 in MESO1 cells, as well as co-modulation of CD9. Addition of anti-CD26 mAb also caused co-modulation of CD9 and CD26 following 48 to 72 h of incubation in the CD26-transfectant MSTO- CD26 (+) cells (Figure 1B). Immunoprecipitation experiments showed that CD26 and CD9 co-precipitated in MESO1 cells and MSTO-CD26 (+) cells (Figure 1C). These results therefore suggest that CD26 and CD9 have physical and potentially functional association.

### The inverse correlation between CD26 and CD9 expression

**Figure 2:**
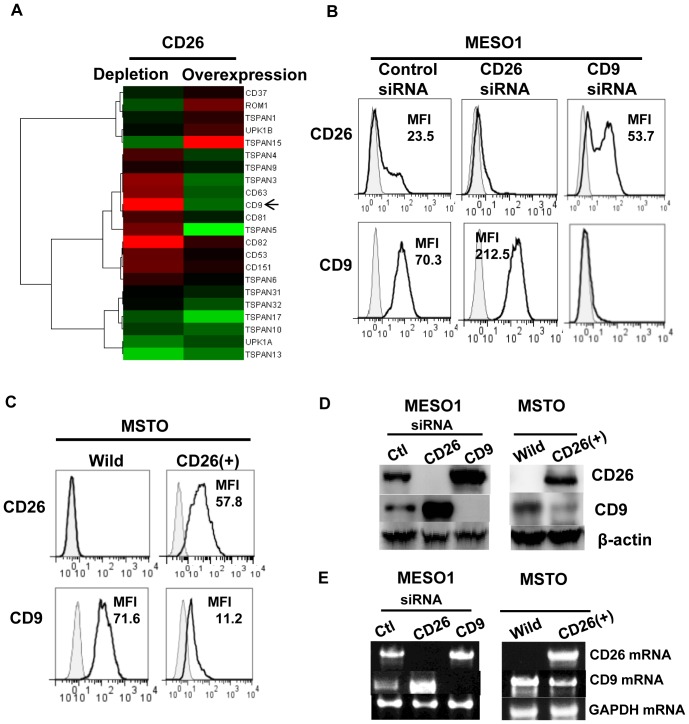
CD26 associates with CD9 in an inverse manner. (A). Heat map representing color-coded expression levels of differentially expressed genes. CD26/Depletion: control siRNA- and CD26 siRNA-transfectedMESO1. CD26/Over expression: MSTO-Wild and MSTO-CD26 (+) cells. Upregulated (red) or downregulated (green). (B and C). MESO1 transfectants of control siRNA, CD26 siRNA, and CD9 siRNA, or MSTO-Wild and MSTO-CD26 (+) cells were stained with anti-CD26-FITC or anti-CD9-FITC and subjected to flow cytometry. (D). MESO1 transfectants with control siRNA, CD26 siRNA, and CD9 siRNA, or MSTO-Wild and MSTO-CD26 (+) cells were lysed and probed with anti-CD26 polyclonal antibody, anti-CD9 mAb (5H9) and anti-β-actin polyclonal antibody. (E). RT-PCR was carried out for analysis of CD26 and CD9 gene expressions on MESO1 transfectants with controlsiRNA, CD26siRNA, and CD9siRNA, or on MSTO-Wild and MSTO-CD26 (+) cells. GAPDH amplification was used as internal control. These results were also confirmed by 5 separate experiments.

In order to understand the nature of the association between CD26 and CD9, we performed microarray analysis of CD26-depleted and CD26 over-expressed mesothelioma cells. Interestingly, CD26 depletion augmented CD9, while CD26 overexpression down-regulated CD9. The expression of other tetraspanins such as TSPAN3-5, and CD63 exhibited similar behavior as CD9, whereas that of TSPAN15 and ROM1 displayed the opposite behavior (Figure 2A).

Since microarray analysis indicated that the expression level of CD26 and CD9 was inversely correlated, we examined the effect of CD26 depletion on CD9, and that of CD9 depletion on CD26 expression. Flow cytometric analysis revealed that depletion of CD26 by CD26 siRNA-1 augmented CD9 surface expression, while depletion of CD9 by CD9 siRNA-1 augmented that of CD26 (Figure 2B). Since similar results were obtained using other set of siRNAs for CD26 or CD9 which were designed to target different sites of the gene (data not shown), CD26 siRNA-1 and CD9 siRNA-1 were used for further study.

We next examined the effect of CD26 overexpression on CD9 expression. Flow cytometric analysis showed that CD26-overexpression down-regulated CD9 in MSTO-CD26 (+) cells (Figure 2C). Immunoblotting analysis also confirmed that the protein level of CD9 was elevated after depletion of CD26 mRNA, and that the level of CD26 protein was elevated after depletion of CD9 mRNA in MESO1 cells (Figure 2D). Furthermore, overexpression of CD26 resulted in down-regulation of CD9 (Figure 2D).

RT-PCR analysis confirmed that depletion of CD26 mRNA augmented CD9 gene expression, while CD9 depletion augmented CD26 gene expression in MESO1 cells (Figure 2E). Moreover, RT-PCR analysis confirmed that overexpression of CD26 reduced CD9 gene expression in MSTO-CD26 (+) cells (Figure 2E).

These results indicate that CD26 functionally associates with CD9 in an inverse manner, and that this association may be regulated at the transcriptional level.

An inverse relationship between CD26 and CD9 was also demonstrated in experiments involving CD26 and CD9 shRNAs. In MESO1 cells transduced with CD26 shRNA-1 or CD9 shRNA-1, an inverse correlation between CD26 and CD9 expression was confirmed (Figure S1A), and similar results were also obtained with CD26 shRNA-2 or CD9 shRNA-2 (data not shown). Similar results were also seen in the mesothelioma cell line NCI-H2452 transduced with CD26 or CD9 siRNA (Figure S1B).

### CD26 potentiates tumor cell invasion

**Figure 3:**
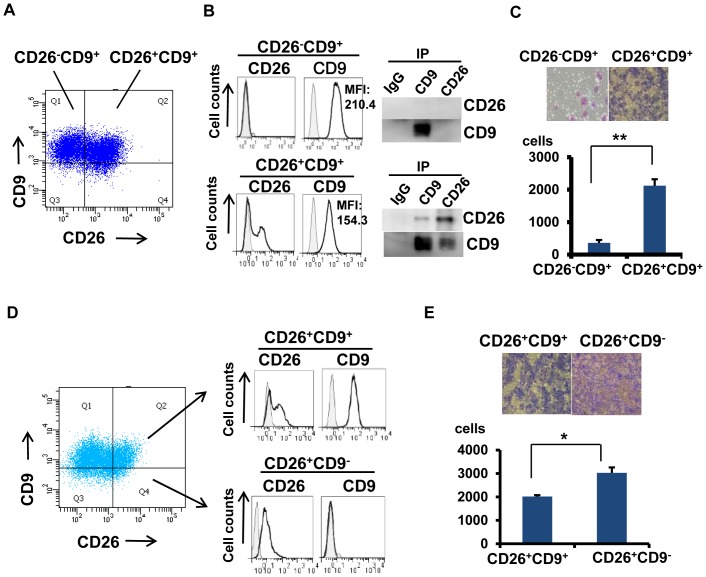
CD26 potentiates tumor cell invasion. (A). Flow cytometric analysis of CD26 and CD9 expressions in MESO1. (B). Sorting of CD26^−^CD9^+^ cells and CD26^+^CD9^+^ cells, and immunoprecipitation was performed with humanized anti-CD26 mAb and anti-CD9 mAb (5H9), then probed with anti-CD26 polyclonal antibody and anti-CD9 mAb (5H9). (C and E).Tumor cell invasion was measured with the Boyden chamber-based cell invasion assay for 24 h. Number of invaded cells was represented as means ± SE (n = 5).*p<0.01, **p<0.001. (D). Flow cytometric analysis of CD26 and CD9 expressions in MESO1 and sorting of CD26^+^CD9^+^ cells and CD26^+^CD9^−^ cells. These results were also confirmed by 3 separate experiments.

We next examined the role of CD26 and CD9 association in cellular biology. While flow cytometric analysis showed that MESO1 cells were composed of CD26^−^CD9^−^, CD26^−^CD9^+^, CD26^+^CD9^+^, and CD26^+^CD9^−^ populations (Figure 3A), CD9 expression on CD26^+^CD9^+^ cell surface was lower than on CD26^−^CD9^+^ cells (Figure 3A). Sorting of CD26^−^CD9^+^ cells and CD26^+^CD9^+^ cells by FACS also showed that surface expression of CD9 on CD26^+^CD9^+^ cells was lower than that on CD26^−^CD9^+^ cells (Figure 3B). Immunoprecipitation experiments showed that CD26 co-precipitated with CD9 in CD26^+^CD9^+^ cells (Figure 3B). Therefore, relatively low expression of CD9 on CD26^+^CD9^+^ cells may reflect the inverse relationship between CD9 and CD26 expression in CD26^+^CD9^+^ cells.

Since invasion is an important aspect of malignancy, we compared invasive potential of CD26^−^CD9^+^ cells and CD26^+^CD9^+^ cells using the Boyden chamber-based cell invasion assay. As shown in Figure 3C, markedly higher invasive potential was observed in CD26^+^CD9^+^ cells in sharp contrast to that of CD26^−^CD9^+^ cells.

We next examined invasive potential of CD26^+^CD9^+^ and CD26^+^CD9^−^ cells (Figure 3D). CD26^+^CD9^+^ and CD26^+^CD9^−^ cells were sorted and invasiveness was determined by the same assay (Figure 3E). CD26^+^CD9^−^ cells showed higher invasion activity than that of CD26^+^CD9^+^ cells, suggesting that CD9 may suppress tumor cell invasion. Previous work involving proteomic analysis of human colon cancer cells by LC-MS/MS identified CD26 as one of the CD9-associated proteins in metastatic cells, but not in the primary tumor cells [30]. Although the exact nature of the CD26 and CD9 association was not elucidated in the report, our current data clearly demonstrate that CD26^+^CD9^+^ cells exhibit higher invasive activity than CD26^−^CD9^+^ cells, suggesting that CD26 may potentiate tumor cell invasiveness.

### CD9 regulates CD26-mediated tumor cell invasive potential

**Figure 4:**
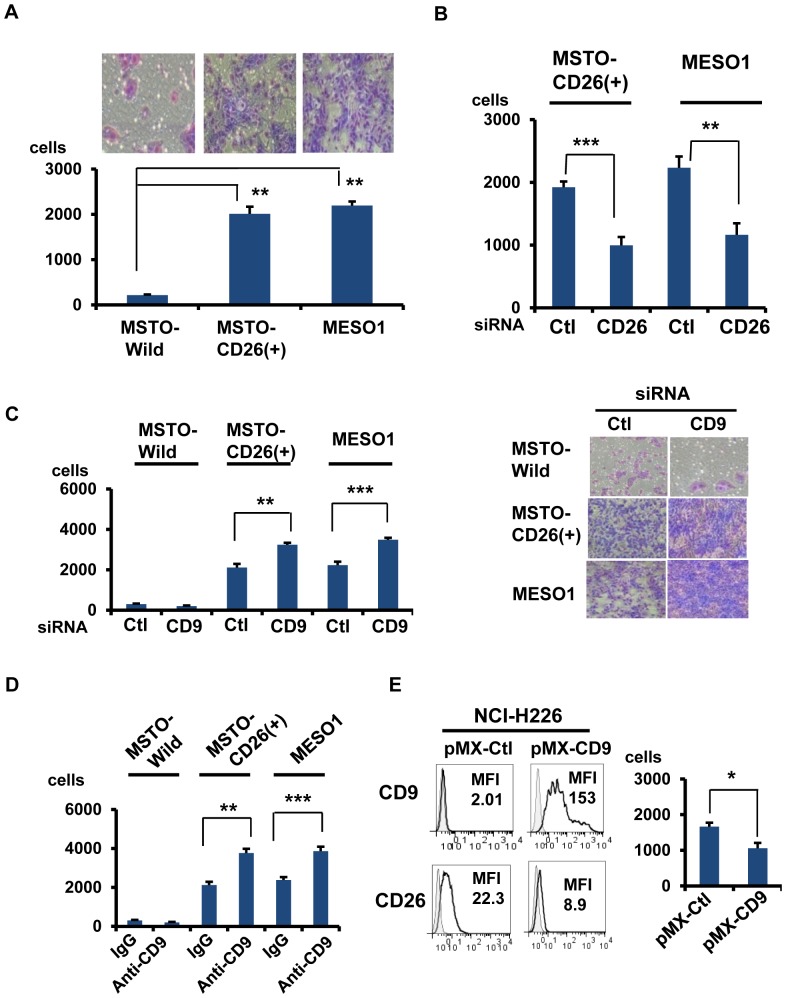
CD9 negatively regulates CD26-mediated invasion. (A-E).Cells were analyzed by the cell invasion assay for 24 h. Number of invaded cells were represented as means ± SE (n = 5). (A). MSTO-Wild,MSTO-CD26 (+), and MESO1 cells. Invaded cells stained are shown in the top panel. **p<0.005. (B). MSTO-CD26 (+) or MESO1 cells transfected with controlsiRNA or CD26siRNA. **p<0.005, ***p<0.001. (C). MSTO-Wild,MSTO-CD26 (+), and MESO1 cells transfected with control-siRNA or CD9siRNA. Invaded cells stained are shown in the right panel. **p<0.005, ***p<0.001. (D). MSTO-Wild, MSTO-CD26 (+), and MESO1 cells treated with control IgG or anti-CD9 mAb (10 µg/ml).**p<0.005, ***p<0.001. (E). NCI-H226 was transfected with pMX vector control or pMX-CD9. After staining with CD26-FITC and CD9-FITC, cells were subjected to flow cytometry.Boyden chamber-based cell invasion assay was performed with NCI-H226 transfected with pMX vector control or pMX-CD9 for 24 h. Number of invaded cells/well was represented as means ± SE (n = 5).*p<0.05.

With our previous data suggesting that CD26 potentiates tumor cell invasiveness, we further evaluated the effect of overexpression and gene depletion of CD26 on invasiveness and motility of MSTO-CD26(+) and MESO1 cells. As shown in Figure 4A, only a small number of MSTO-Wild cells invaded as compared to a markedly greater number of MSTO-CD26 (+) cells. MESO1 cells which endogenously express CD26 also showed higher level of invasiveness than MSTO-Wild cells (Figure 4A). Meanwhile, this high level of invasiveness was reduced following gene knockdown of CD26 with siRNA (Figure 4B). Similar results were obtained with the cell migration assay (Figure S2A and S2B). These results indicate that CD26 may confer greater tumor cell invasiveness and motility. On the other hand, down-regulation of CD9 by siRNA augmented invasion and migration of MSTO-CD26 (+) and MESO1 cells, but not MSTO-Wild cells (Figure 4C and Figure S2C). In view of previous data suggesting CD9 involvement in suppressing tumor metastasis [17], CD26 ability to promote tumor cell invasion and migration may be influenced by the anti-metastatic activity of CD9. Anti-CD9 mAb treatment consistently enhanced invasiveness and motility of MSTO-CD26 (+) and MESO1 cells, but not MSTO-Wild cells (Figure 4D and Figure S2D). Furthermore, overexpression of CD9 on the CD26-positive mesothelioma cell line NCI-H226 decreased CD26 expression and suppressed invasion (Figure 4E).

Taken together, these results suggest that CD9 regulates migration and invasion of CD26-positive cells by its inhibitory effect on CD26-mediated invasiveness.

### CD26 potentiates invasiveness through its association with α5β1 integrin

We next investigated the molecular mechanism involved in CD26 ability to enhance invasiveness of mesothelioma cells. Multiple reports demonstrated that β1 integrins have key roles in cancer metastasis and invasion [31]. Among α1 to α6 integrins, α5 and α6 integrins were detected on the surface of MESO1 and MSTO- CD26 (+) cells. Meanwhile, treatment with anti-CD26 mAb led to co-modulation of α5β1, but not α6β1 integrin, in MESO1 and MSTO-CD26 (+) cells (data not shown).

**Figure 5:**
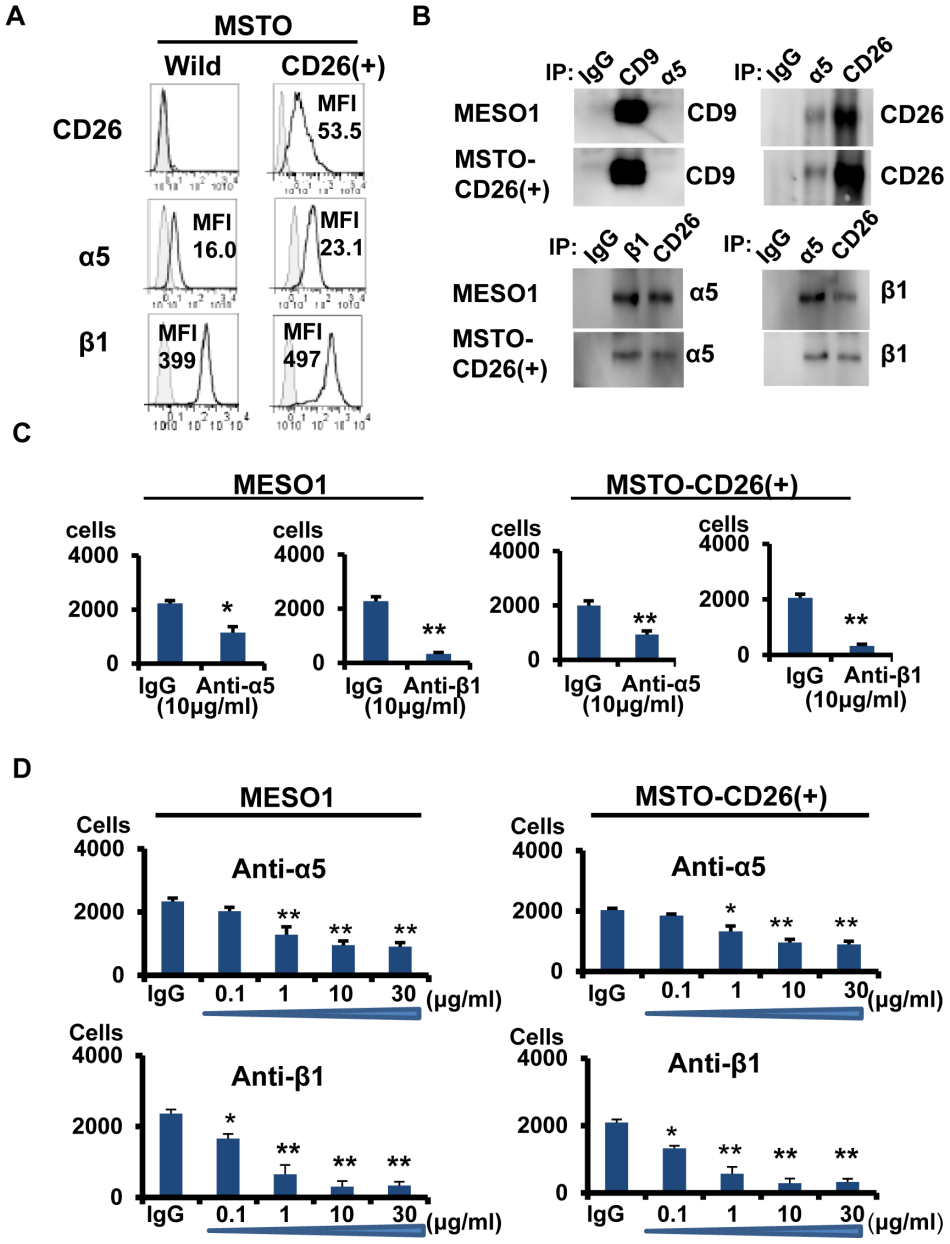
CD26 potentiates invasiveness through α5β1 integrin. (A).MSTO-Wild, MSTO-CD26 (+) cells were subjected to flow cytometry for CD26, α5, and β1. (B). MESO1 and MSTO-CD26 (+) cells were subjected to immunoprecipitation with control IgG, humanized anti-CD26 mAb, anti-CD9 mAb (5H9), anti-α5 mAb (2H6), or anti-β1 mAb (4B4). The immunoblot was probed with anti-CD26 polyclonal antibody, anti-CD9 mAb (5H9), anti-α5 mAb (2H6), or anti-β1 mAb (4B4). (C). Boyden chamber-based cell invasion assay of MESO1 and MSTO-CD26 (+) cells treated with anti-α5, and β1 antibodies for 24 h. (n = 5). *p<0.01, **p<0.005. (D).The cell migration assay of MESO1 and MSTO-CD26 (+) cells treated with anti-α5, and β1 antibodies.(n = 5). *p<0.05, **p<0.01.

Additional analysis of the interaction between α5β1 integrin and CD26 showed that transfection of CD26 cDNA into MSTO-Wild cells increased the level of α5β1 expression (Figure 5A). Immunoprecipitation analysis indicated that α5β1 integrin co-precipitated with CD26, but not with CD9 in MESO1 and MSTO-CD26 (+) cells (Figure 5B). These data therefore suggest that CD26 associates with α5β1 integrin in mesothelioma cells.

We next studied the effect of antibodies against α5 and β1 on invasiveness according to previously described methods [26]. Treatment with anti-integrin α5 or β1 mAb inhibited tumor cell invasion and migration of MESO1 and MSTO-CD26 (+) cells (Figure 5C and 5D). Therefore the present results indicate that α5β1 integrin may play an important role in CD26-mediated promotion of migration and invasion in mesothelioma cells.

### Down-regulation of CD9 enhances invasiveness through its inverse relationship with CD26

**Figure 6:**
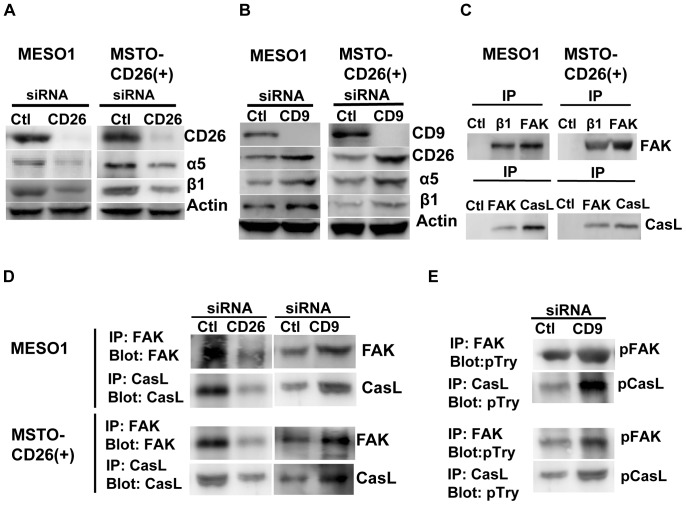
Downregulation of CD9 enhances CD26-mediated invasive potential. (A). MESO1 and MSTO-CD26 (+) cells transfected with control siRNA and CD26-siRNA were subjected to immunoblotting using anti-α5 (2H6), anti-β1 (4B4) mAbs, anti-CD26 polyclonal antibody, anti-β-actin polyclonal antibody. (B). The same cells transfected with control-siRNA and CD9-siRNA. Anti-CD9 mAb (5H9) was used for immunoblotting. (C). MESO1 and MSTO-CD26 (+) cells were subjected to immunoprecipitation to anti-β1 mAb (4B4), anti-FAK mAb (10G2), and anti-Cas-L Ab (TA248). Immunoblotting was performed with anti-FAK (10G2), and anti-Cas-L Ab (TA248). (D). MESO1 and MSTO-CD26 (+) cells were transfected with control siRNA, CD26 siRNA or CD9 siRNA, then subjected to immunoprecipitation with anti-FAK mAb (10G2), or anti-Cas-L Ab (TA248). Immunoblotting was performed with anti-FAK mAb (10G2) or anti-Cas-L Ab (TA248). (E). MESO1 and MSTO-CD26 (+) transfectants with control siRNA or CD9 siRNA were immunoprecipitated with anti-FAK mAb (10G2) or anti-Cas-L Ab (TA248). Immunoblotting was performed with anti-phosphotyrosine mAb (4G10). Similar results were observed by 3 separate experiments.

We next evaluated the effects of CD9 down-regulation on invasiveness of CD26-positive cells. SiRNA-mediated CD26 depletion of CD26-positive cells led to reduced α5β1 integrin level as detected by immunoblotting (Figure 6A). These results confirm that CD26 potentiates tumor cell invasiveness through its regulation of α5β1 integrin. On the other hand, siRNA-mediated depletion of CD9 augmented the expression of CD26 and α5β1 integrin, as detected by immunoblotting (Figure 6B). Since CD9 itself does not associate with α5 integrin (Figure 5B), it is plausible that down-regulation or perturbation of CD9 may augment CD26 expression in an inverse manner, and the resultant increased level of the CD26-α5β1 complex likely contributes to enhanced invasive potential. Since CD9 overexpression led to decreased CD26 expression and suppression of tumor cell invasion (Figure 4E), we next determined the effect of CD9 overexpression on α5β1 integrin expression. On CD9-overexpressed NCI-H226 cells, the expression of α5β1 integrin was reduced together with that of CD26 (data not shown). Therefore, the opposing effect of CD9 and CD26 on migration and invasive potential is mediated at least partly through their effect on α5β1 integrin expression.

We previously demonstrated that Crk-associated substrate lymphocyte type (Cas-L)/human enhancer of filamentation 1 (HEF1)/neural precursor cell expressed, developmentally down-regulated 9 (NEDD9) mediates cell signaling through β1 integrins [32]–[34], and that Cas-L promotes metastasis of non-small cell lung cancer cells [35]. We therefore examined the possible involvement of Cas-L in CD26-mediated mesothelioma cell invasiveness. Immunoprecipitation experiments indicated that β1 associates with focal adhesion kinase (FAK), and FAK with Cas-L in MESO1 and MSTO-CD26 (+) cells (Figure 6C). CD26 depletion by siRNA resulted in decreased expression of FAK and Cas-L, while CD9 depletion augmented FAK and Cas-L level in MESO1 and MSTO-CD26 (+) cells (Figure 6D). These results hence suggest the involvement of FAK and Cas-L in CD26-mediated invasiveness.

Since CD9 depletion led to increased CD26 expression (Figure 2B, Figure 6B, and Figure S1) and promoted invasiveness and motility (Figure 4C and Figure S2C), we evaluated the effect of CD9 depletion on tyrosine phosphorylation levels of FAK and Cas-L. As shown in Figure 6E, CD9 depletion led to increased FAK and Cas-L tyrosine phosphorylation in mesothelioma cells, indicating that CD26 and CD9 may regulate the overall protein level and tyrosine-phosphorylation of β1 integrin-related signaling molecules as well as the expression of α5β1 integrin.

### Combined treatment with anti-CD26 mAb and anti-CD9 mAb on tumorigenesis

**Figure 7:**
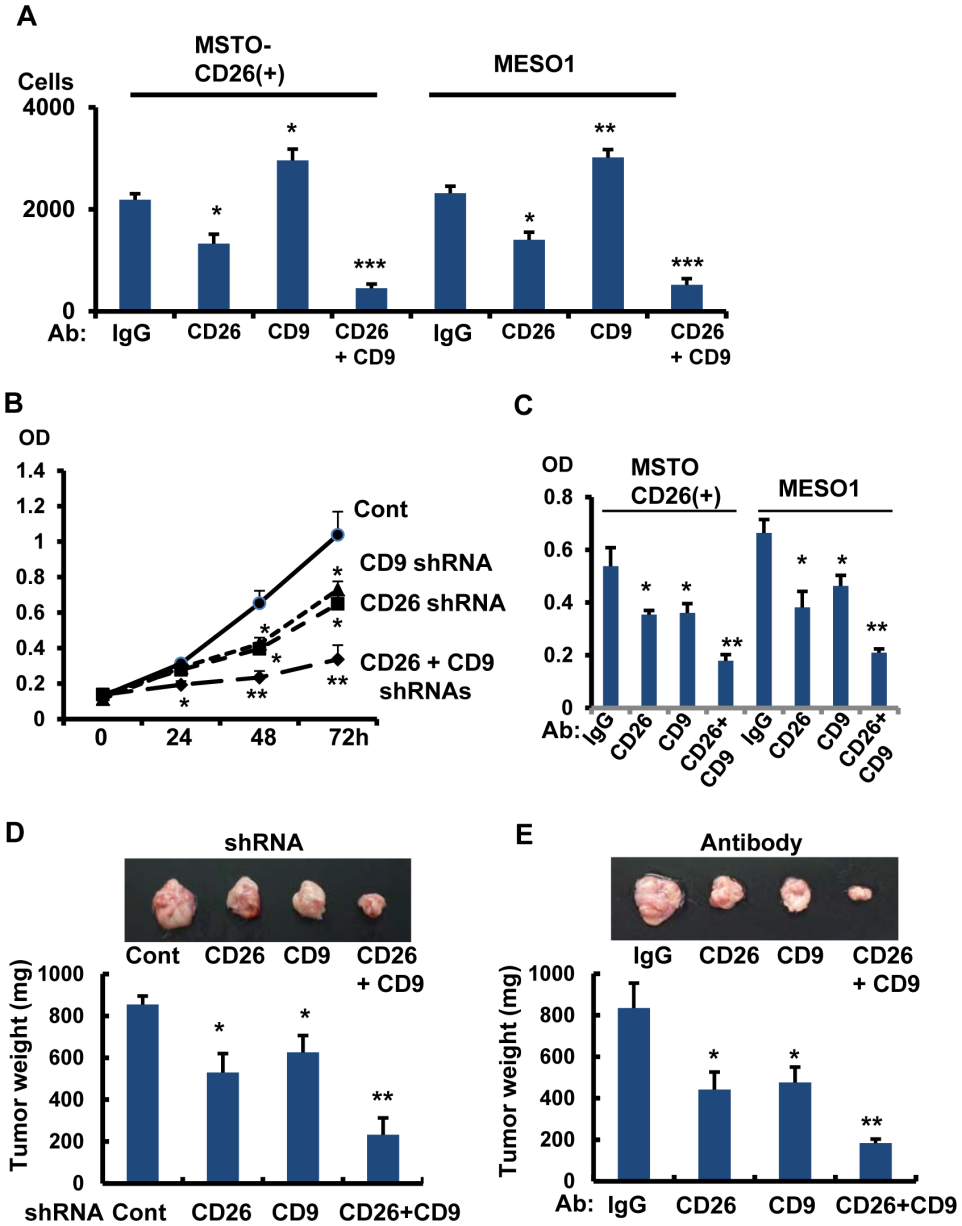
Combined treatment with anti-CD26 mAb and anti-CD9 mAb on tumorigenesis. (A). MSTO-CD26(+) and MESO1 cells were treated with anti-CD26 mAb (10 µg/ml), anti-CD9 mAb (10 µg/ml), or with anti-CD26 mAb (5 µg/ml) + anti-CD9 mAb (5 µg/ml). Cell invasion assay was performed at 24 h. Number of invaded cells was represented as means ± SE (n = 5). *p<0.05, **p<0.01, ***p<0.005. (B). MESO1 cells transfected with shRNAs for control, CD26, CD9, or CD26-CD9 were grown in 96 well culture plates and subjected to MTT assay at indicated times. Each data point represents the mean ± SE of six wells. *p<0.05, **p<0.01. (C). MSTO-CD26(+) and MESO1 cells were treated with anti-CD26 mAb (10 µg/ml), anti-CD9 mAb (10 µg/ml), or with anti-CD26 mAb (5 µg/ml) + anti-CD9 mAb (5 µg/ml). MTT assay was performed at day 2. Each data point represents mean ± SE of six wells. *p<0.05, **p<0.005. (D). SCID mice were inoculated with MESO1 cells transfected with shRNAs for control, CD26, CD9 or CD26-CD9. Tumors were sampled at day 14. Tumor weight was represented as means ± SE (mg) among 5 tumors from each category. The representative tumor images were shown on the top. *p<0.05, **p<0.01. (E).MESO1 cells were implanted into SCID mice and intraperitoneally treated with anti-CD26 mAb (8 mg/kg), anti-CD9 mAb (8 mg/kg), or with anti-CD26 mAb (4 mg/kg) + anti-CD9 mAb (4 mg/kg) 2 times in a week from the day following tumor implantation. Tumors were sampled at day 14. Tumor weight was represented as means ± SE (mg) among 5 tumors from each category. The representative tumor images were shown in the top. *p<0.05, **p<0.01.

Our present study indicated that blocking of CD26 expression by gene-depletion inhibited tumor cell invasiveness. Since CD26 interacts with CD9 in an inverse correlation, it is plausible that combined blocking of both CD26 and CD9 may effectively inhibit tumor cell invasiveness. We then examined the effect of combined blocking of CD26 and CD9 with their respective antibodies. In MSTO-CD26(+) cells, anti-CD26 mAb inhibited tumor cell invasiveness. As shown in Figure 4D, anti-CD9 mAb treatment enhanced invasiveness (Figure 7A). However, combined treatment with anti-CD26 mAb and anti-CD9 mAb markedly inhibited invasiveness (Figure 7A). Similar results were obtained in MESO1 cells (Figure 7A).

We next examined the effects of combined blocking of CD26 and CD9 on tumor cell proliferation. CD26-depletion with shRNA inhibited in vitro tumor cell growth of MESO1 cells (Figure 7B). In contrast to the enhanced effects on invasiveness by CD9-depletion, gene-depletion of CD9 inhibited tumor growth (Figure 7B). Furthermore, combined depletion of both CD26 and CD9 effectively inhibited in vitro tumor growth (Figure 7B). Similarly, blocking of CD26 or CD9 by antibodies inhibited in vitro tumor growth in MSTO-CD26(+) and MESO1 cells (Figure 7C). Moreover, combined treatment with anti-CD26 mAb and anti-CD9 mAb resulted in enhanced inhibition of tumor growth in these cell lines (Figure 7C). In mouse xenograft study, depletion of CD26 or CD9 by shRNA inhibited in vivo tumor growth (Figure 7D), and combined depletion of CD26 and CD9 caused prominent suppression of tumor growth in vivo (Figure 7D). Similarly, anti-CD26 mAb or anti-CD9 mAb inhibited tumor growth in vivo. Furthermore, combined treatment with anti-CD26 mAb and anti-CD9 mAb resulted in marked inhibition of tumor growth in vivo. Therefore, combined blockade of both CD26 and CD9 might be a potential therapeutic approach for malignant mesothelioma.

## Discussion

In the present study, we propose a novel molecular mechanism that involves the reciprocal interaction of CD26 and CD9. We first demonstrated antibody-induced co-modulation and co-precipitation of CD9 and CD26. At the transcriptional level, expression of CD9 and CD26 mRNAs was regulated in an inverse manner. Meanwhile, these molecules exhibited opposite effects on invasion and migration of mesothelioma cells, with CD26 having an enhancing effect and CD9 showing a suppressing effect. These contrasting effects of CD9 and CD26 were further elucidated by gene knockdown and gene transfer experiments. Furthermore, we demonstrated co-precipitation of CD26 and α5β1 integrin adhesion receptor, which partially explains the pro-metastatic behaviors of CD26^+^ tumor cells. Finally, perturbation of CD26 or CD9 expression resulted in changes of the overall protein and tyrosine-phosphorylation levels of FAK and Cas-L, the β1 integrin-mediated signaling molecules involved in cell adhesion and migration.

Tetraspanins form complexes by interacting with other tetraspanins as a homodimer or a heterodimer and with other classes of molecular species that are required for their biological function [24]. Molecular compensation is one of the characteristics of tetraspanins. Depletion of individual tetraspanin which forms embryonic synapse in Drosophila results in minor defects in development due to the overlapping expression of tetraspanins with similar functions, which compensates for the absence of another tetraspanin [36]. Compared with single knockdown of these tetraspanins, depletion of two or three tetraspanins markedly decreased cancer cell invasion, due to the overlapping contributions of these tetraspanins [37]. These molecular compensations are supported by overlapping expression of tetraspanins with similar functions. In contrast to the reported molecular compensations based on overlapping expressions of tetraspanins, our current work demonstrates an association between tetraspanin and non-tetraspanin molecules to regulate their expressions in an inverse manner. This kind of association has been recently reported in the case of CD9 and CD9P-1, in which the expression of CD9P-1 positively correlates with the metastatic status of lung tumor cells [38]. In the present study, we demonstrated that inverse correlation between CD9 and CD26 play a role on CD9-mediated suppression of invasiveness of CD26-positive tumor cells.

We previously reported the localization of CD26 in lipid raft and the association between CD26 and caveolin-1, a molecule residing in the lipid raft [5] and caveolae [6]. An association between α5β1 integrin and caveolin-1 has been reported to be necessary for integrin-mediated Shc-Ras-ERK signaling [39], and that interaction between phospho-caveolin-1 and integrins reversibly regulates the internalization of lipid raft [40]. Despite proposed differences in the biochemical properties and molecular contents of TEM and lipid raft [24], [41], it should be noted that CD26 has been preferentially detected in TEM of metastatic colon cancer cells [30], data which partially support our present findings. Although the precise distribution of CD26, CD9, and integrins in these membrane microdomains remains unclear, but warrant examination.

Metastasis is the critical feature of malignancy which influences overall survival of patients [42]. In human colon cancer, CD26 was identified as a novel marker for cancer stem cells, and injection of CD26^+^ cells into SCID mice resulted in the development of distant metastasis, indicating the metastatic capacity of the CD26^+^ cells [43]. Reduced expression of CD9 correlates with enhanced metastasis in many types of malignancies [17], suggesting that CD9 predominantly functions as a suppressor of metastasis. Consistent with these finding was our recent multivariate analysis showing that CD9 expression is an independent favorable prognostic marker of malignant mesothelioma [44].

Antibodies against α5β1 integrins inhibited cell invasion and migration, and depletion of CD26 concomitantly reduced the expression of α5β1 integrin. We therefore conclude that CD26 promotes invasiveness through the formation of CD26-α5β1 integrin molecular complex. On the other hand, depletion of CD9 augmented both α5β1 integrin and CD26 expression, resulting in enhanced level of the CD26-α5β1 integrin complex. Our results differ from previous work indicating that downregulation of CD9 correlates with decreased levels of α5 and β1 integrins, contributing to dissemination of ovarian carcinomas [45]. This discrepancy may be partly attributable to differences in cellular origin and molecular contents of tetraspanins or CD26.

Several molecular mechanisms involved in CD9-mediated suppression of metastasis have been reported, including modification of β1 integrin [46] and inhibition of WAVE2 [47]. In the present study, we show that CD9 suppresses cell invasion and migration by inhibiting the formation of CD26-α5β1 integrin complex through its negative regulation of CD26. Our results therefore suggest a new mechanism involved in CD9-mediated suppression of invasiveness and metastasis.

Based on the above findings, blocking of both CD26 and CD9 resulted in marked inhibition of invasiveness and proliferation of tumors. Therefore, combined application of anti-CD26 and anti-CD9 mAb is likely a promising therapeutic strategy for malignant mesothelioma.

In conclusion, our present study demonstrates that the interaction between CD26 and CD9 mediates mesothelioma behavior, while suggesting that CD26 and CD9 would be promising biomarkers as well as molecular targets for the future treatment of malignant mesothelioma.

## Supporting Information

Figure S1 **Negative correlation of CD26 and CD9 expression.** (A). MESO1 cells transfected with control shRNA, CD26 shRNA-1, and CD9 shRNA-1 were stained with anti-CD26-FITC or with anti-CD9-FITC, and subjected to flow cytometry. (B). NCI-H2452 cells transfected with control-siRNA, CD26-siRNA, and CD9-siRNA were also analyzed by CD26 and CD9-FITC.
(TIF)

Figure S2 **CD26 potentiates migration, and negative regulation by CD9.** (A and B). Migration of MESO1, MSTO-Wild, and MSTO-CD26 (+) cells, or MESO1 or MSTO-CD26 (+) cells transfected with control siRNA or CD26 siRNA were analyzed by the Boyden chamber-based cell migration assay, for 24 h. Number of migrated cells/well was represented as means ± SE (n = 5).*p<0.005, **p<0.001. (C).Migration of MSTO-Wild, MSTO-CD26 (+), and MESO1 cells transfected with control siRNA or CD9 siRNA were analyzed. Number of migrated cells/well was represented as means ± SE.(n = 5).*p<0.005. (D) Migration of MSTO-Wild, MSTO-CD26 (+), andMESO1 cells treated with control IgG or anti-CD9 mAb (5H9) were analyzed. Number of migrated cells/well was represented as means ± SE.(n = 5).*p<0.05, **p<0.005.
(TIF)

Checklist S1 **Checklist for mice in vivo xenograft study.** Combined treatment with humanized anti-CD26 mAb and anti-CD9 mAb on mice in vivo tumor growth.
(DOCX)
