# Supplementary figures and images for: CD9 Negatively Regulates CD26 Expression and Inhibits CD26-Mediated Enhancement of Invasive Potential of Malignant Mesothelioma Cells
Source: PLoS One. 2014 Jan 23;9(1):e86671. doi: 10.1371/journal.pone.0086671 (PMC3900581; doi:10.1371/journal.pone.0086671)

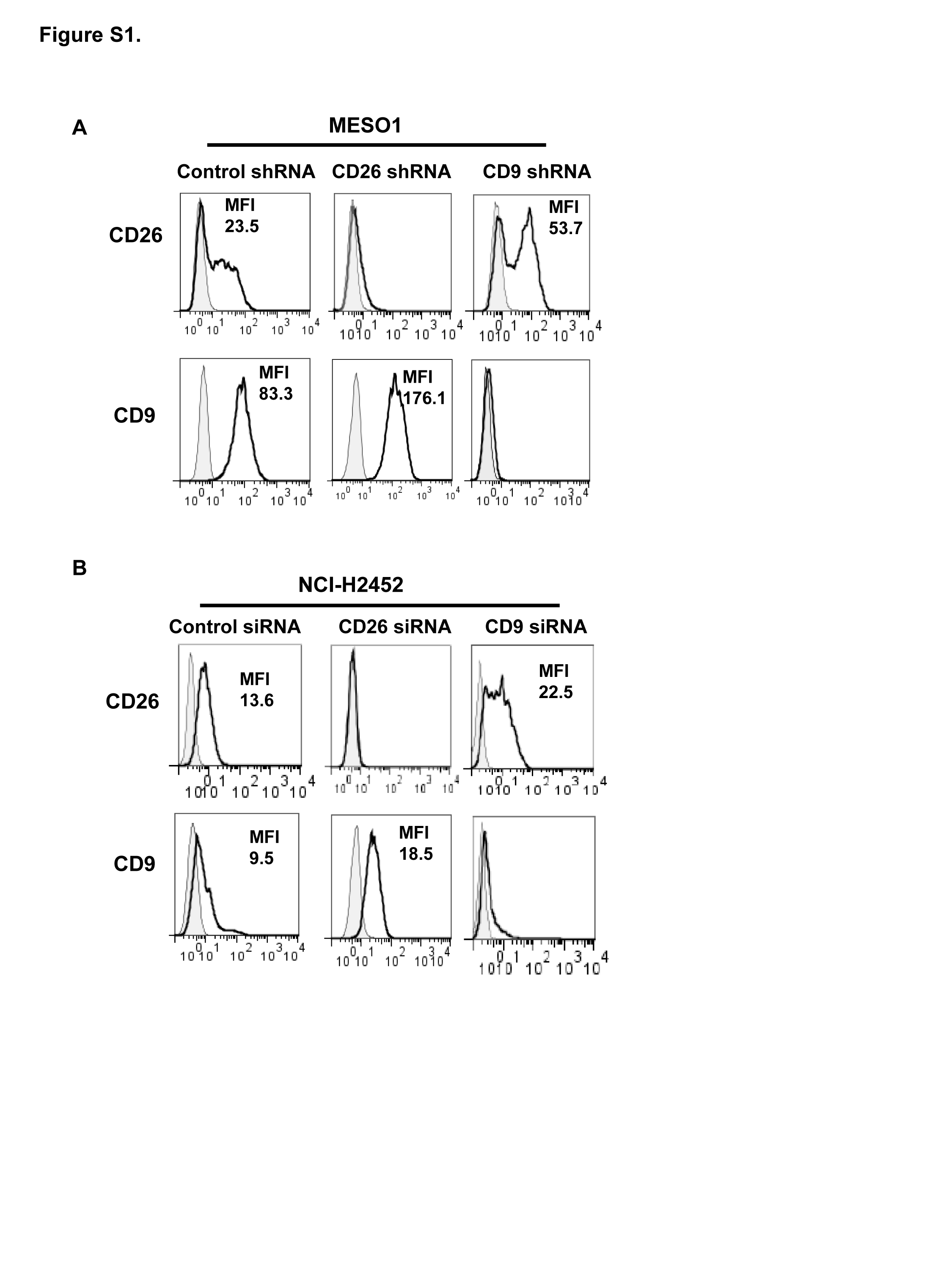

Supplement: Figure S1 — Negative correlation of CD26 and CD9 expression. (A). MESO1 cells transfected with control shRNA, CD26 shRNA-1, and CD9 shRNA-1 were stained with anti-CD26-FITC or with anti-CD9-FITC, and subjected to flow cytometry. (B). NCI-H2452 cells transfected with control-siRNA, CD26-siRNA, and CD9-siRNA were also analyzed by CD26 and CD9-FITC. (TIF) [file pone.0086671.s001.tif]

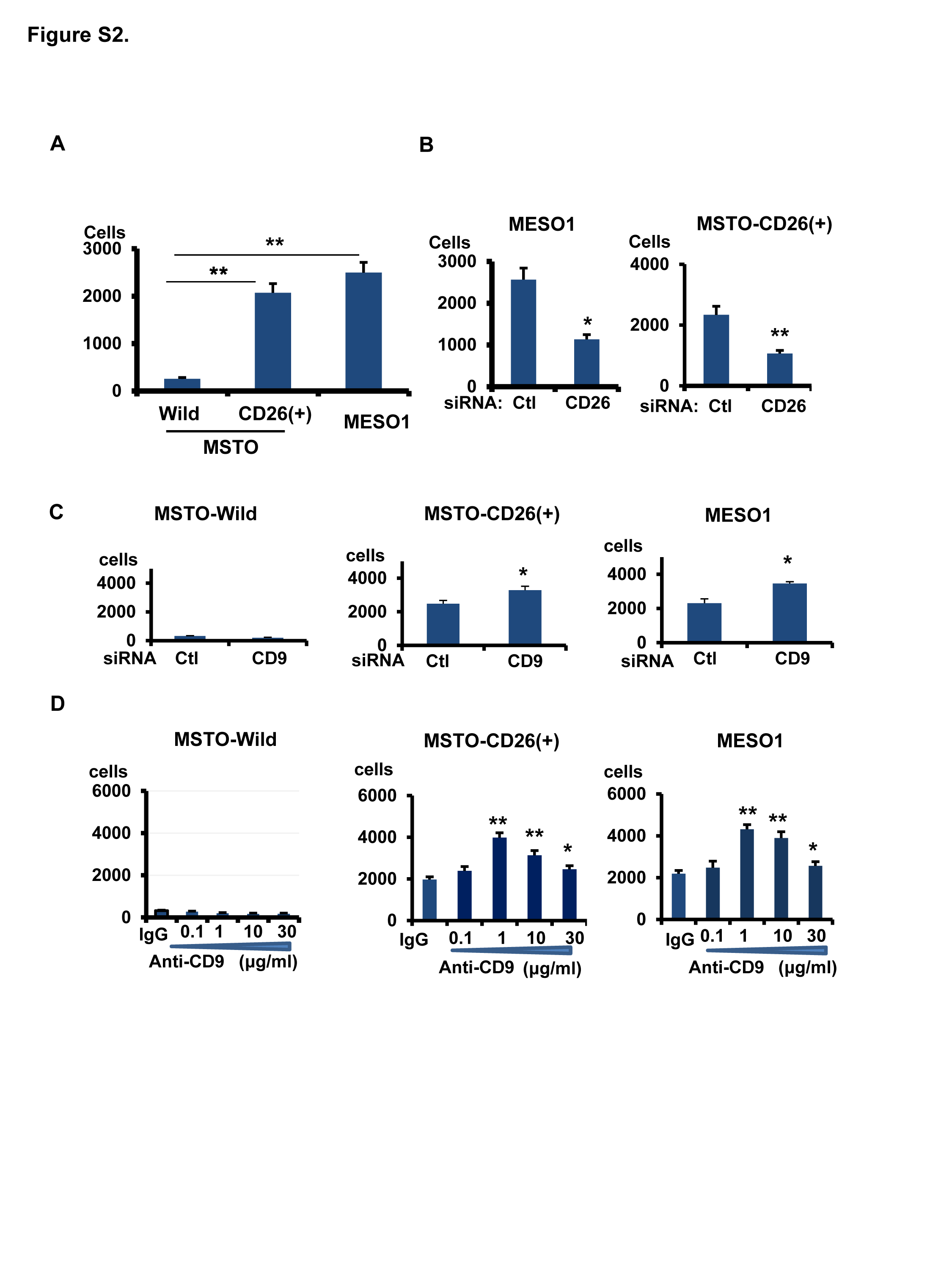

Supplement: Figure S2 — CD26 potentiates migration, and negative regulation by CD9. (A and B). Migration of MESO1, MSTO-Wild, and MSTO-CD26 (+) cells, or MESO1 or MSTO-CD26 (+) cells transfected with control siRNA or CD26 siRNA were analyzed by the Boyden chamber-based cell migration assay, for 24 h. Number of migrated cells/well was represented as means ± SE (n = 5).*p<0.005, **p<0.001. (C).Migration of MSTO-Wild, MSTO-CD26 (+), and MESO1 cells transfected with control siRNA or CD9 siRNA were analyzed. Number of migrated cells/well was represented as means ± SE.(n = 5).*p<0.005. (D) Migration of MSTO-Wild, MSTO-CD26 (+), andMESO1 cells treated with control IgG or anti-CD9 mAb (5H9) were analyzed. Number of migrated cells/well was represented as means ± SE.(n = 5).*p<0.05, **p<0.005. (TIF) [file pone.0086671.s002.tif]
