# Supplementary material for: CD9 Negatively Regulates CD26 Expression and Inhibits CD26-Mediated Enhancement of Invasive Potential of Malignant Mesothelioma Cells
Source: PLoS One. 2014 Jan 23;9(1):e86671. doi: 10.1371/journal.pone.0086671 (PMC3900581; doi:10.1371/journal.pone.0086671)
Supplement: Checklist S1 — Checklist for mice in vivo xenograft study. Combined treatment with humanized anti-CD26 mAb and anti-CD9 mAb on mice in vivo tumor growth. (DOCX) [file pone.0086671.s003.docx]

**Checklist S1**

**Mice in vivo xenograft study**

**1. Title**

Combined treatment with humanized anti-CD26 mAb and anti-CD9 mAb on mice in vivo tumor growth

**2. Abstract**

In in vitro study, blocking of CD26 or CD9 inhibited mesothelioma cell growth. Furthermore, combined blocking of CD26 and CD9 resulted in efficient suppression of tumor growth compared with single blockade of CD26 and CD9. For clinical implications, we attempted to study the combined blockade of both CD26 and CD9 on tumor growth in vivo. We performed the study using female SCID mice. In the first experiment, mice were implanted with mesothelioma cells which were transfected with CD26 and CD9 shRNAs. In the second experiment, following tumor cell implantation into mice, effects of humanized anti-CD26 mAb and anti-CD9 mAb treatment was examined. Combined blockade of both CD26 and CD9 resulted in enhanced suppression of tumor growth in vivo, compared with single blocking of CD26 or CD9. Therefore, combined treatment of both humanized anti**-**CD26 mAb and anti**-**CD9 mAb might be a promising therapeutic strategy for malignant mesothelioma.

**3. Background**

a. Molecular compensation is a characteristic of tetraspanins and depletion of two or three tetraspanins markedly decreased cancer invasion (Lafleur et al Mol Biol Cell 20:2030, 2009). In the present study, CD26 and CD9 interact in an inverse relationship. Therefore, it is speculated that blockade of both CD26 and CD9 may result in enhanced suppression of tumor growth.

b. For therapeutic implications in the clinical setting, in addition to in vitro studies, we examined this issue in in vivo studies. Since mouse xenograft model is a standard method to examine therapeutic agents or antibodies, we performed the study in a mouse xenograft model.

**4. Objective**

In in vitro studies, combined blockade of both CD26 and CD9 resulted in marked

inhibition of tumor growth compared with single blockade of CD26 or CD9. Thus, we attempted to examine the effects of combined blockade of both CD26 and CD9 in a mouse xenograft model.

**5. Ethical statement**

All experiments using mice were approved by and carried out following the guidelines of the Institute Animal Care and Use Committee of the University of Tokyo (Tokyo, Japan),

**6. Study design**

a. In the first study, effects of combined blockade of both CD26 and CD9 were examined on 4 groups of mice by implanting mesothelioma cells transfected with shRNAs for control, CD26, CD9 or CD26+CD9. In the second study, mice were implanted with mesothelioma cells and examined in 4 groups which were treated with control IgG, humanized anti-CD26 mAb, anti-CD9 mAb, or humanized anti-CD26 and CD9 mAbs.

b. In order to minimize the effects of subjective bias when allocating mice, we allocated mice to make the average body weights as equal as possible among the experimental groups

c. One experimental group involved 5 mice, and was accommodated in one separate cage.

**7. Experimental procedure**

a. Mice were implanted with tumor cells under ether anesthesia. Effective amount of ether to produce anesthesia was presented on a paper towel laid on the bottom of a small chamber. The paper towel is covered with another paper towel in order to avoid direct contact of mice with the paper presented with ether. Mouse was put into the chamber. After anesthesia was induced, the mouse was removed from the chamber. Dorsal region of the mouse was wiped with cotton wetted with 70% alcohol. Then, tumor cells (5 x 10^5^/100 µl PBS/mouse) were subcutaneously implanted into the dorsal region. In the study treating mice with antibodies, antibodies were dissolved in PBS and intraperitoneally administered into mice in a volume of 100 µl. At study completion, mice were sacrificed by inhalation with carbon dioxide.

b. Implantation of tumors or antibody treatment was performed at 9:00 am.

c. Animal study was done at the Laboratory Animal Research Center of the Institute of Medical Science of the University of Tokyo.

d. In order to protect mice from noxious stimuli by anesthetic injection through needle, ether anesthesia was chosen. In antibody treatment, effects of antibodies on tumor growth was examined in the preliminary study at the dose of 3, 8, 10, and 30 mg/kg, and antibodies at the dose of 8, 10 and 30 mg/kg significantly inhibited in vivo tumor growth. In order to minimize loading to mice, the dose of 8 mg/kg of antibody was chosen. Carbon dioxide euthanasia was chosen to sacrifice the mice, since it requires a short time period without noxious stimuli.

**8. Experimental animal**

a. Female SCID mice 5 weeks age were obtained from CHARLES RIVER LABORATORIES JAPAN, Inc. Mice were used before reaching their developmental stage of 8 weeks of age. Mean mouse weight was around 20g.

b. SCID (Severe Combined Immunodeficiency) mice possess a genetic autosomal recessive mutation designated *scid*. This mutation was first detected in 1980 by Dr.M.J.Bosma and his associates in a congenic strain (C.B-17/Icr) of mice at the Fox Chase Cancer Center in Philadelphia, Pennsylvania. SCID mice are homozygous (C.B-17/Icr scid/scid) for the scid gene which has been mapped to the centromeric end of chromosome 16. These animals are coisogenic with the normal C.B-17/Icr +/+ strain.

**9. Housing and husbandry**

a. Mice were housed in cages with specific pathogen free facilities.

b. A 12 hour light/12 hour dark cycles was used. Temperature between 18 and 23^o^C, and humidity 40-60% were kept. Mice were given adlibitum access to food and water. Mouse facility was kept clean at all times to prevent pollution of the environment by microorganisms, etc.

c. Welfare-related assessments and interventions were carried out routinely by the Institute Animal Care and Use Committee of the University of Tokyo (Tokyo, Japan),

**10. Sample size**

a. In the first study in which mice were implanted with shRNA-transfected tumor cells, total of 20 mice were used. The experiment involved 4 groups with 5 mice in each group. In the second study in which mice were treated with antibodies after tumor cell implantation, total of 20 mice were used. The experiment involved 4 groups with 5 mice in each group.

b. 5 mice were used as the least number of animals per experimental group to ascertain the reproducibility of the experimental results, as commonly conducted in mouse in vivo study..

**11. Allocating animals to experimental groups**

a. Allocation of mice was performed in the same way in the first and second studies. The weights of all the mice were measured, and mice were allocated into 4 groups with 5 mice in each group. The mice were allocated in such a way as to make the verage weight of mice to be equal among the 4 groups as much as possible.

b. In the first study in which shRNA-transfected tumor cells were implanted, shRNA-transfected tumors were implanted in the following order of the groups; control-shRNA transfected tumors, CD26 shRNA-transfected tumors, CD9 shRNA-transfected tumors, CD26 and CD9 shRNAs-transfected tumors. In the second study in which mice were treated with antibodies after tumor cells were implanted, antibodies were administered in the following group orders; control IgG, anti-CD26 mAb, anti-CD9 mAb, humanized anti-CD26 and CD9 mAbs.

**12. Experimental outcome**

Experimental outcome was defined by the tumor weight.

**13. Statistical method**

a. Statistical analysis was performed by two-tailed *t*-test.

b. One group with 5 mice was set as a unit of analysis.

c. p<0.05 was considered as statistically significant.

**14. Basement data**

Prior to tumor implantation or treatment, no problematic characteristics or health status of mice was observed.

**15. Numbers analyzed**

Five mice in each group were included in each analysis (100%).

**16. Outcomes**

In the first study, tumor weight (mg) of the mice implanted with tumor cells transfected with shRNAs were Control shRNA (855±37, n=5), CD26 shRNA (536±88, n=5, p<0.05), CD9 shRNA (626±80, n=5, p<0.05), and CD26+CD9 shRNAs (233±76, n=5, p<0.01).

In the second study, tumor weight (mg) of the mice treated with antibodies were Control IgG (835±120, n=5), humanized anti-CD26 mAb (442±84, n=5, p<0.05) , anti-CD9 mAb (476±74, n=5, p<0.05), and humanized anti-CD26+CD9 antibodies (184±19, n=5, p<0.01).

**17. Adverse events**

a. There was no adverse event in each experimental group.

b. No modification to the experimental protocols was made to reduce adverse events.

**18. Interpretation/scientific implication**

a. It was hypothesized that combined blocking of both CD26 and CD9 may enhance inhibitory effects of tumor growth, and this hypothesis was validated by this study.

b. No limitation is considered on this study.

c. It is preferred that the number of mice used be reduced to as few as possible.

**19. Generalisability/translation**

Combined treatment of both humanized anti-CD26 mAb and anti-CD9 mAb resulted in enhanced suppression of tumor growth in mice. Therefore, this combined treatment with humanized anti-CD26 mAb and CD9 mAb might be a promising therapeutic strategy for malignant mesothelioma.

**20. Funding**

This work was supported by grants-in aid from the Ministry of Education, Science, Sports and Culture, and Ministry of Health, Labor and Welfare, Japan (No.24659401 C.Morimoto), and by the Program for Promotion of Fundamental Studies in Health Sciences of the National Institute of Biomedical Innovation (No.07-17 C.Morimoto). These founders play a role to contribute materials.
